# Validity of the French version of Catquest-9SF and use of an electronic notepad for entering patient-reported outcome measures

**DOI:** 10.1186/s40662-021-00233-7

**Published:** 2021-04-01

**Authors:** Gregory Katz, Alexandra Rouquette, François Lignereux, Thierry Mourgues, Michel Weber, Mats Lundström

**Affiliations:** 1 Chair of Innovation & Value in Health, University of Paris School of Medicine, Paris, France; 2 Value-Based Health Care Consortium, Paris, France; 3 Prom-Time, Paris, France; 4 Service de Santé Publique et d’Épidémiologie, AP-HP Paris-Saclay, Le Kremlin-Bicêtre, France; 5 Centre de recherche en Épidémiologie et Santé des Populations, Inserm, Université Paris-Saclay, Villejuif, France; 6 Ophtalmologie Chénieux, Polyclinique de Limoges, Limoges, France; 7 Institut Ophtalmologique Sourdille-Atlantique, Elsan Santé Atlantique, Nantes, France; 8 Ophthalmology Department, Centre Hospitalier-Universitaire de Nantes, Nantes, France; 9 Department of Clinical Sciences, Ophthalmology, Faculty of Medicine, Lund University, Lund, Sweden

**Keywords:** Cataracts, Patient-related outcome measures, Quality of life, Questionnaire, Value-based health care, Catquest-9SF

## Abstract

**Background:**

The Catquest-9SF questionnaire is a patient reported outcome measure that quantifies the visual benefits from cataract surgery. The purpose of this study was to translate and adapt the Catquest-9SF questionnaire for France, to assess its psychometric properties via Rasch analysis, and to assess its validity when completed using an electronic notepad.

**Methods:**

The Catquest-9SF questionnaire was translated following the guidelines of the International Society for Pharmacoeconomics and Outcomes Research. Catquest-9SF and clinical data were collected from patients before and after routine cataract surgery. All questionnaire data were collected via an electronic notepad. Rasch analysis was performed to assess psychometric properties, and sensitivity to change was analysed for patients with complete paired pre- and post-operative questionnaires.

**Results:**

A complete filled-in preoperative questionnaire was obtained for 848 patients. Rasch analysis showed good precision (person separation: 2.32, person reliability: 0.84), ordered category probability curves, no item misfit, and unidimensionality. The respondents were slightly more able than the level of item difficulty (targeting: −1.12 logits). Sensitivity was analysed on 211 paired questionnaires, and the postoperative questionnaires showed a clear ceiling effect. The effect size was 2.6. The use of an electronic notepad for completing the questionnaire worked out very well after some adjustments.

**Conclusions:**

The French version of Catquest-9SF has good psychometric properties and is suitable for use in French-speaking patients. The use of the Catquest-9SF questionnaire in an electronic format showed good validity.

**Supplementary Information:**

The online version contains supplementary material available at 10.1186/s40662-021-00233-7.

## Background

Cataract surgery is one of the most frequently performed surgical procedures and has low complication rates [1]. While objective measures such as visual acuity and residual refractive error are critical results from the surgeon’s perspective, the ability to perform day-to-day tasks is an essential outcome from the patient’s perspective. Some patients who have undergone uneventful cataract surgery with gains in visual acuity may still not be satisfied [2]. This can be due to a variety of reasons, such as patient expectations, or because visual acuity does not reflect the patient’s quality of life, ability loss, or limitations in daily activities [3]. In addition, the mean waiting time for cataract surgery in OECD countries is 129 days [4], which can cause dissatisfaction and frustration, and strengthens the need to assess patients’ quality of life.

Several patient-reported outcome questionnaires have been developed to quantify the subjective visual function in cataract patients [5]. The Catquest-9SF questionnaire is one such example, measuring activity limitations in daily life for patients with cataract or following cataract surgery [6]. It comprises nine questions: two global items evaluating the patient’s general perception of difficulties and satisfaction with vision, and seven items related to difficulties in performing specific daily activities [6]. Each item has four response options ranging from 1 (very great difficulty) to 4 (no difficulty), and a “cannot decide” option which is treated as a missing answer. A raw score ranging from 9 to 36 is computed in summing the answers to the 9 items. Catquest-9SF has demonstrated robust psychometric properties, and when compared to 15 other Rasch-refined cataract questionnaires it proved to be the most responsive to cataract surgery [7].

The European Registry of Quality Outcomes for Cataract and Refractive Surgery (EUREQUO) database uses Catquest-9SF to assess cataract outcomes and develop evidence-based guidelines for cataract and refractive surgery in Europe [8]. The International Consortium for Health Outcomes Measurement (ICHOM) has adopted Catquest-9SF as a patient-reported outcome measure for cataracts to facilitate international comparisons that can drive improvements in patients’ functional recovery and quality of life [9]. The questionnaire is available in Swedish [10], Australian English [11], German [12], Italian [13], Spanish [14], Dutch [15], Danish [16], and Chinese [17], and is currently being translated into several other languages.

The purpose of the present study was twofold: first, to translate and culturally adapt the Catquest-9SF questionnaire into French and to assess its psychometric properties via Rasch analysis; and second, to assess the validity of Catquest-9SF when completed using electronic notepads. Until now, most patients have used pencil and paper to complete the Catquest-9SF questionnaire, after which the data are entered into a spreadsheet. This spreadsheet data entry is not only tedious and time-consuming, but also carries the risk of typographical errors affecting data quality. These difficulties have stifled the adoption of the Catquest-9SF in daily clinical practice. Validation of an electronic method would enable the immediate use of the patient-reported outcomes during consultations.

## Methods

### Translation

The Catquest-9SF questionnaire was translated from the English version into French by Mapi Language Services (Lyon, France). To optimize both semantic and conceptual equivalence between the source and target language versions, the ten steps of the International Society for Pharmacoeconomics and Outcomes Research (ISPOR) principles of good practice for the translation and cultural adaption process for patient-reported outcomes were adopted [18]. These steps were: preparation, two parallel independent forward translations from English to French, reconciliation, backward translation, backward translation review, harmonization, cognitive debriefing with five native French speakers who had undergone cataract surgery, review of the cognitive debriefing results and finalization, proofreading results and the final report.

### Data collection

Following the French translation of the Catquest-9SF, patients undergoing cataract surgery from February 2018 to August 2019 in two reference centres in France (Institut Ophtalmologique Sourdille-Atlantique in Nantes, and Ophtalmologie Chénieux at the Polyclinique de Limoges) were invited to complete the questionnaire before surgery and within 3 months following surgery. Patients were instructed by their surgeon to fill in the postoperative questionnaire after wearing their new spectacles (if any), and to schedule a postoperative visit when they were used to their new visual status. Exclusion criteria included difficulty with the French language or comprehension, being under guardianship or curatorship, and being under 18 years of age. All patients had a complete ophthalmic examination before and after surgery, and the following data were collected: age, gender, uncorrected distance visual acuity, spherical equivalent refraction, and spectacle-corrected distance visual acuity.

Information about the questionnaire was provided to the patients through the notepad. The patients were only able to access the questionnaire after explicitly confirming that they were not opposed to the scientific use of the data collected through the questionnaire. The dissemination of the questionnaire was systematic, and was part of routine care. These prospectively collected data were used to assess the psychometric properties of the questionnaire.

A declaration to the French data protection authority was registered in 2017 to comply with data pseudonymization and the General Data Protection Regulation (GDPR).

### Digital notepad

**Fig. 1:**
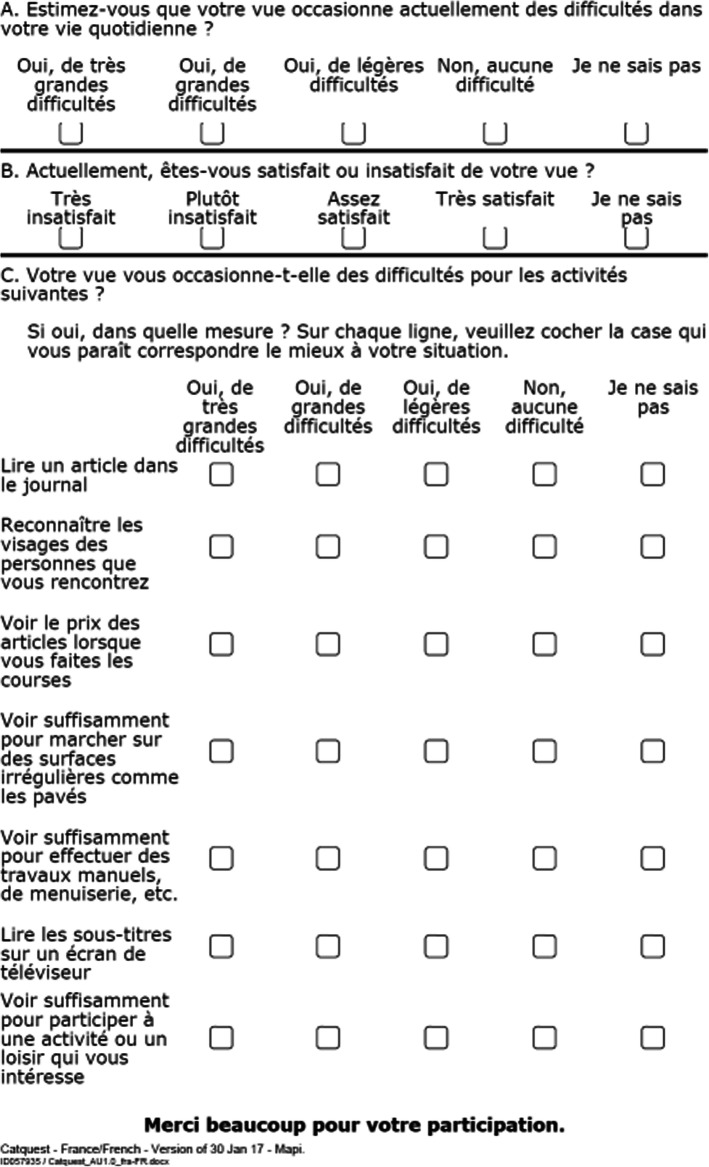
French version of Catquest-9SF

The French version of Catquest-9SF is shown in Fig. 1.

**Fig. 2:**
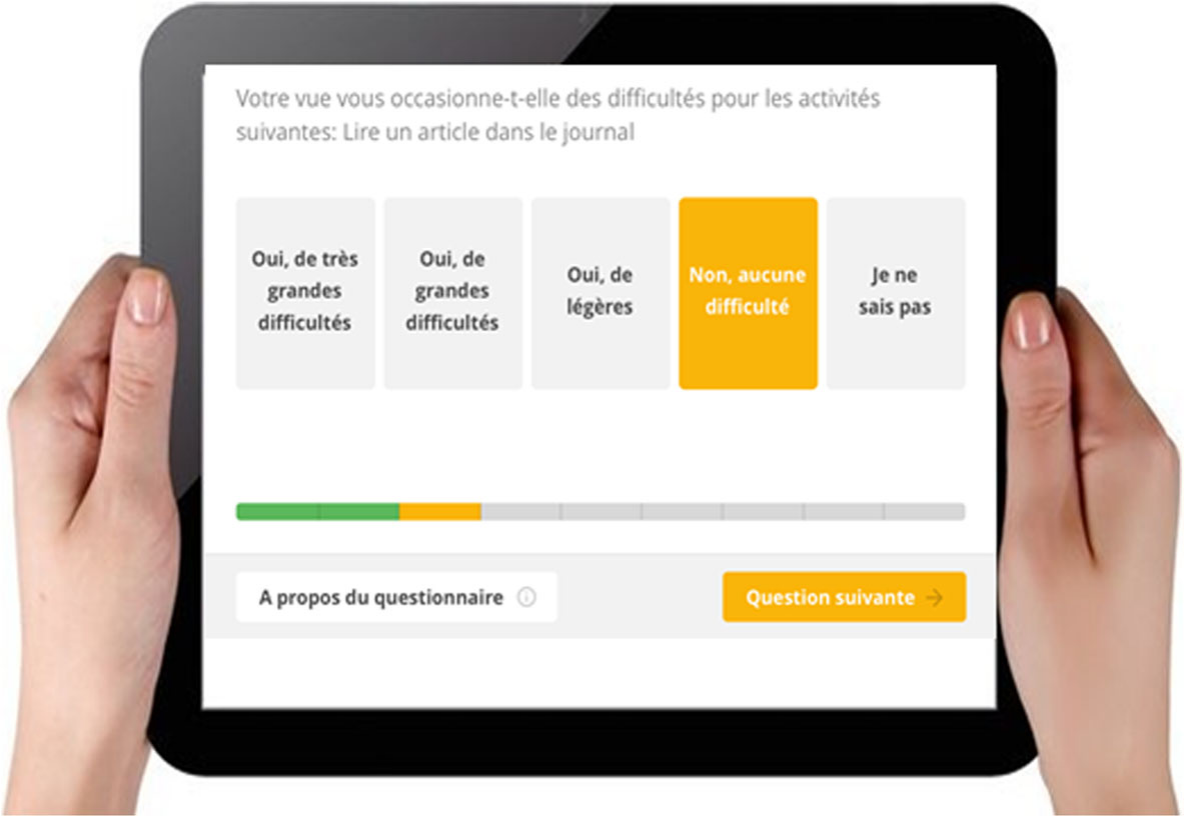
Digital version of the French Catquest-9SF. One question is displayed per screen and large buttons are used for responses

The French version of the Catquest-9SF was integrated into a digital application specifically developed by Value2Health for cataract patients, with one question per screen (Fig. 2). The ergonomic design included a simple layout with large buttons to enable self-reported assessment. All data collection was performed on an electronic notepad. The workflow description of using the notepad can be found in the online supplemental material.

**Fig. 3:**
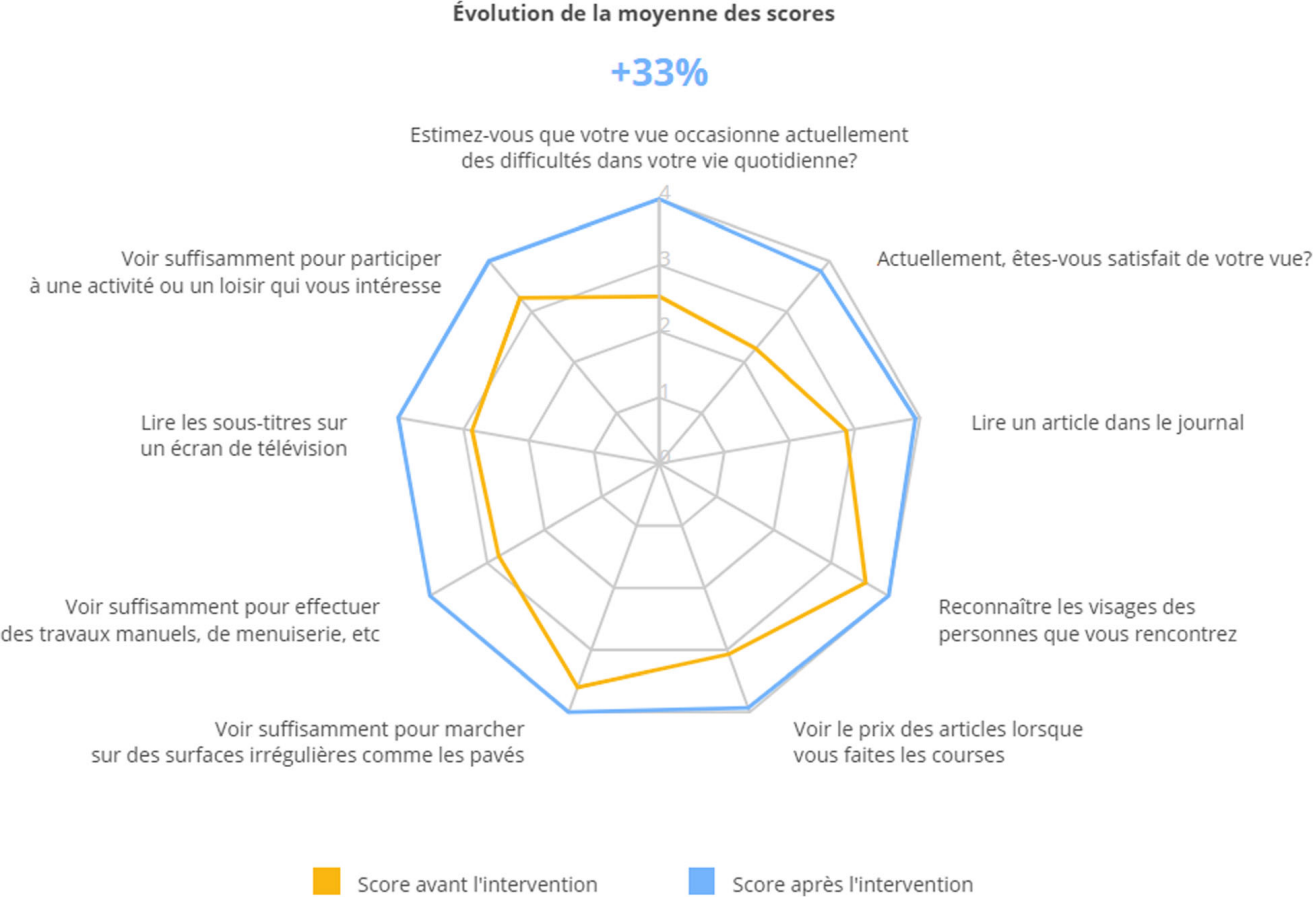
Practitioner’s dashboard with the Catquest-9SF nine items before (in yellow) and after surgery (in blue). One question is given per axis, from question A at the top moving clockwise to question C7. The item score is represented on each axis from zero at the centre of the graph to 4 at the periphery. The evolution in scores from before to after surgery is expressed in percentage (in blue at the center of the dashboard)

The result of a completed Catquest-9SF is visible on the practitioner’s dashboard (Fig. 3).

### Statistical analyses

Analyses were performed on pre-surgical data except for the study of sensitivity to change which used paired pre- and post-surgical data. To be included in the analyses, patients had to respond to both general items (A and B) and at least five of the remaining seven (C1–C7) Catquest-9SF items. Continuous and categorical variables were described as median (1st quartile–3rd quartile) and count (percentage) respectively. Descriptive analyses were conducted using version 15 of Stata [19], and Rasch analysis was performed using version 4.2.0 of Winsteps (Chicago, IL, USA).

Ceiling and floor effects were identified for each item of the Catquest-9SF; these two effects were defined a priori as more than 95% of respondents selecting the highest and the lowest category, respectively [20]. At the scale level, floor and ceiling effects were considered if more than 15% of respondents achieved the lowest or higher possible score, respectively [21]. Reliability was assessed using ordinal Cronbach’s alpha [22] and considered satisfactory if ≥ 0.7 [23]. Sensitivity to change was evaluated using a two-sided paired t-test comparing post- and pre-surgical Catquest-9SF raw scores, with a type-1 error risk set at 0.05. The effect size was also calculated based on Rasch scores.

The characteristics of the Catquest-9SF data were assessed by Rasch analysis, which is based on modern test theory and results in the transformation of an ordinal raw score into an interval Rasch score [24]. This means that the respondents’ ability and the difficulty of the items are evaluated along the same scale. The unit of the scale is the logit, and so both respondents and items are given a logit value which constitutes their Rasch score. The distance in logits between one respondent and one item reflects the probability that the respondent will endorse the item. This probabilistic relationship is the basis for the estimation of interval scores.

Rasch analysis provides extensive insight into the psychometric properties of a questionnaire. This study assessed measurement precision in terms of person separation (> 2.0 considered adequate) and separation reliability (> 0.80 considered adequate), whether response categories are utilized in ascending order (category ordering with category probability curves), item fit to the model (outfit and infit statistics, with 0.70–1.30 considered ideal and 0.50–1.50 acceptable), and whether the scale measures one or more dimensions (i.e., dimensionality; for unidimensionality, ≥ 60% of variance explained by the measure is ideal, while ≥ 50% is acceptable; the eigenvalue of the first contrast should be < 2.0). Assessment of psychometric performance followed the standards of Khadka et al. [5].

## Results

Of the 894 patients included during the 18-month recruitment period, 848 (331 males, 39%) responded to items A and B and at least 5 of the remaining 7 Catquest-9SF items before the surgery. Their median age was 71.9 (67.6–78.2) years. One-eye surgery was performed in 388 (46%) of these patients while the remainder underwent both-eye surgery. The distribution of the pre-surgical responses to the nine Catquest-9SF items is shown in Table 1. The percentage of missing answers (or “Cannot decide” option) was always lower than 5%, and there was no ceiling or floor effect in any of the nine items. The median pre-surgical Catquest-9SF raw score was 27 (23–30), and there was no ceiling or floor effect at the scale level. The reliability was satisfactory, with a Cronbach alpha coefficient of 0.87.

**Table 1:** Responses to the nine items of the pre-surgery Catquest-SF9

Item, N (%)	1-Very great difficulty	2-Great difficulty	3-Some difficulty	4-No difficulty	Total responses	Missing
A - Difficulties in any way in daily life	44 (5)	252 (30)	469 (55)	83 (10)	848 (100)	0 (0)
B - Satisfaction with vision^a^	102 (12)	476 (56)	250 (30)	20 (2)	848 (100)	0 (0)
C1 - Reading text in newspaper	68 (8)	232 (27)	377 (44)	169 (20)	846 (100)	2 (0)
C2 - Recognizing faces	24 (3)	98 (12)	239 (28)	483 (57)	844 (100)	4 (0)
C3 - Seeing prices	41 (5)	191 (23)	365 (43)	235 (28)	832 (98)	16 (2)
C4 - Seeing to walk on uneven ground	18 (2)	105 (12)	342 (40)	379 (45)	844 (100)	4 (0)
C5 - Seeing to do needlework, etc.	44 (5)	180 (21)	364 (43)	222 (26)	810 (96)	38 (4)
C6 - Reading text on TV	90 (10)	253 (30)	347 (41)	150 (18)	840 (99)	8 (1)
C7 - Seeing to carry out a preferred hobby	22 (3)	123 (14)	342 (40)	327 (49)	814 (96)	34 (4)

^a^Response modalities: 1-Very dissatisfied, 2-Fairly dissatisfied, 3-Fairly satisfied, 4-Very satisfied

The Rasch analysis showed good precision, with a person separation of 2.32 and a person reliability of 0.84. The category probability curves were ordered for both kinds of response options (A&C1–7 and B; see Table 1 footnote). Item statistics showed no misfit, meaning no redundancy and no outliers among the items. The fit statistics included infit and outfit mean square, and both values should be close to 1 for the items. In this study, the infit mean square varied between 0.70 and 1.32 and the outfit mean square between 0.74 and 1.25, thus lying in an ideal range (Table 2).

**Table 2:** Fit statistics and Rasch measure of item difficulty for the Catquest-9SF items

Item	Infit mean square	Outfit mean square	Rasch measure
A - Difficulties in any way in daily life	0.70	0.74	−0.60
B - Satisfaction with vision	1.00	1.00	−2.21
C1 - Reading text in the newspaper	1.08	1.07	−0.40
C2 - Recognizing faces	1.32	1.25	1.59
C3 - Seeing prices	0.81	0.81	0.13
C4 - Seeing to walk on uneven ground	1.14	1.12	1.18
C5 - Seeing to do needlework etc.	0.96	0.94	0.12
C6 - Reading text on TV	1.14	1.13	−0.70
C7 - Seeing to carry out a preferred hobby	0.94	0.87	0.88

**Fig. 4:**
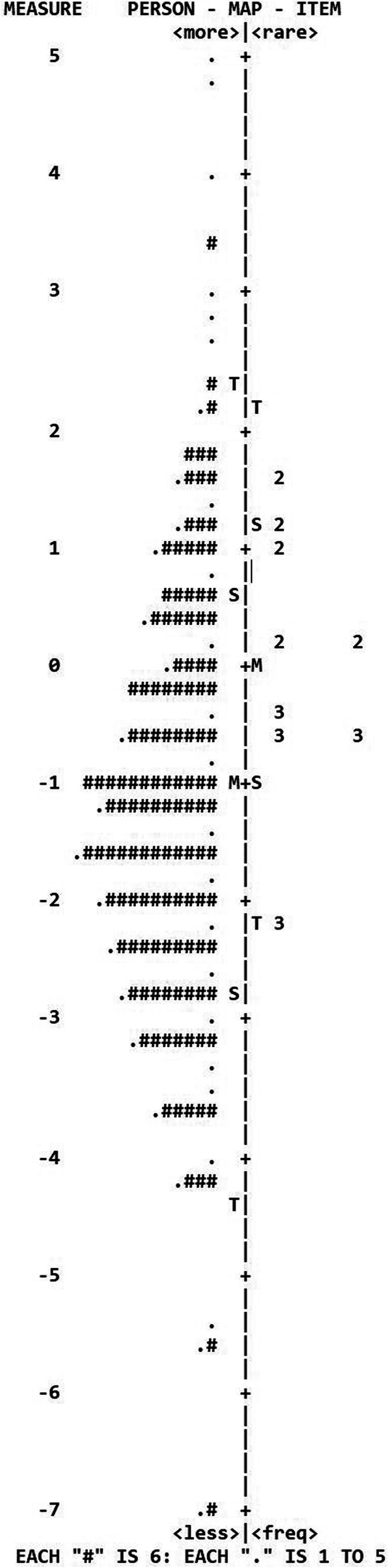
Person-item map for preoperative data. Items to the right, 2 or 3 signifies the 9 items, and persons to the left, # signifies 6 persons. M stands for mean value, S for 1 standard deviation and T for 2 standard deviations

The observed raw variance explained by the measures was 58% and the unexplained variance in the first contrast had an eigenvalue of 1.60, indicating a unidimensional questionnaire. Differential item functioning was not observed for gender in any item (< 0.5 logits in all cases). The targeting (item difficulty versus person ability) for preoperative data showed a mean person Rasch score of −1.12 (item score set to 0), indicating that the respondents were more able than the difficulty of the items. This is also shown in the person-item map (Fig. 4).

**Fig. 5:**
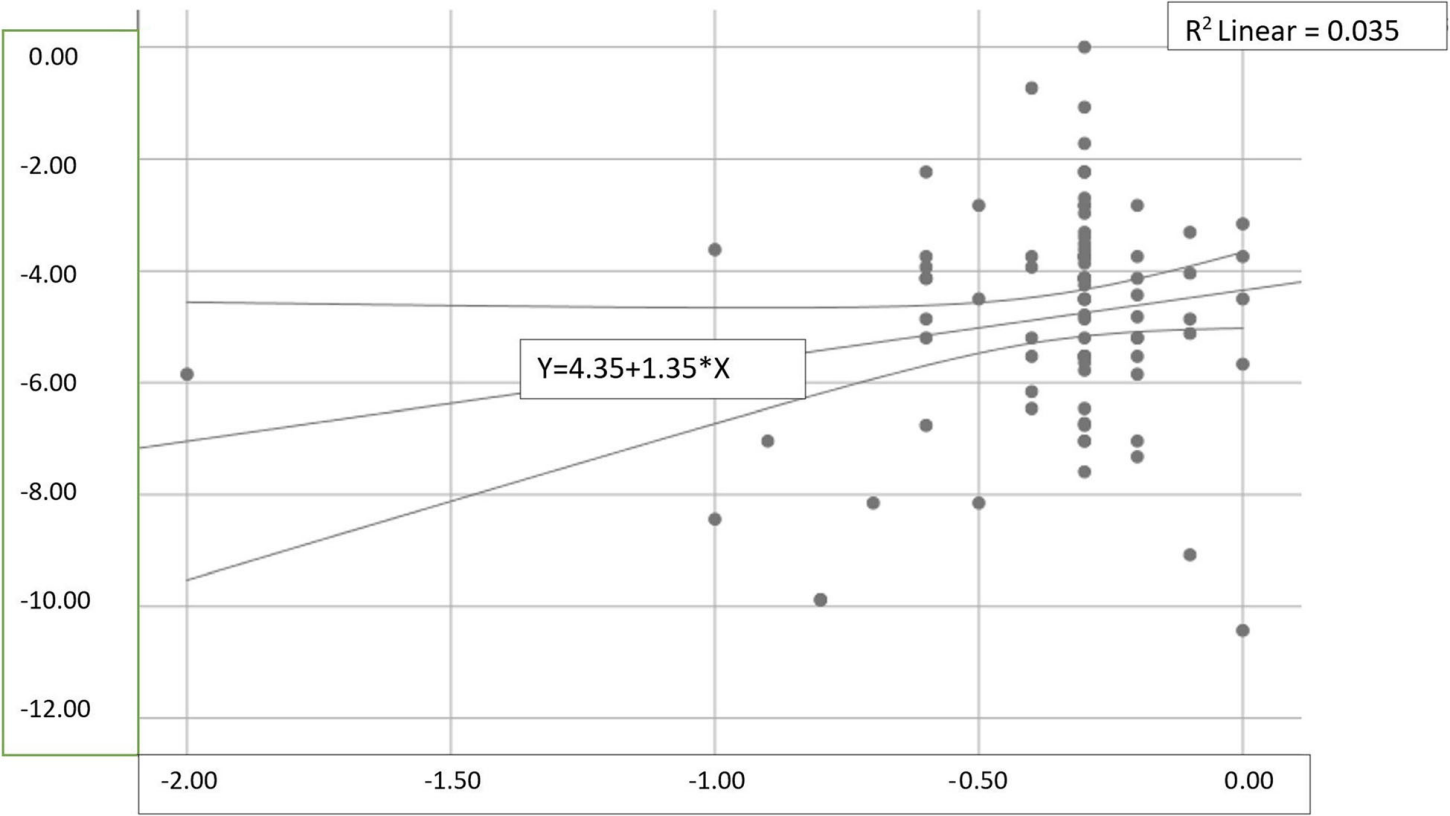
Scatterplot with improvement after surgery in Rasch score vs. improvement in visual acuity (logMAR) after surgery. Y-axis: Rasch score improvement in logits, X-axis: Visual acuity improvement, in logMAR units

The Catquest-9SF questionnaire was filled in by 211 patients during the 3 months after surgery (mean number of days after surgery: 26 ± 21 days). A clear ceiling effect was present in this post-surgical assessment, with 132 (62.6%) patients showing the highest possible raw score. The median raw score changes from pre- to post-surgical assessment was 8 (5–11) points, and the paired t-test showed a significant difference between the pre- and post-surgical raw scores (*P* < 0.0001). Rasch analysis of the 211 completed pre and postoperative questionnaires showed a large improvement in the average person Rasch score, from −1.16 ± 1.73 logits to −5.72 ± 1.24 logits. The improvement was on average 4.56 ± 2.02 logits. The effect size calculated as improvement/standard deviation for baseline was 2.6. The improvement in Rasch score after surgery vs. the improvement in visual acuity (logMAR notation) is demonstrated in a scatter plot (Fig. 5).

## Discussion

In this study, the nine items of the French version of Catquest-9SF completed using an electronic notepad were shown to be Rasch homogenous and to have good psychometric properties. The Cronbach alpha coefficient and the person separation and person reliability indices were congruent and indicated an adequate reliability for the entire scale. The questionnaire was also found to be sensitive to change. As shown in Fig. 4, there was a relationship between improvement in Rasch score (logits) and improvement in visual acuity (logMAR) after surgery.

In daily clinical practice, scientific evidence is lacking in identifying patients who are likely to significantly benefit from cataract surgery [25]. The information from Catquest-9SF can be valuable during patient consultations as it helps to determine the optimal time of intervention [26]. It may also help to explain the progression of the disease and to manage the patient’s expectations [27]. The data generated by Catquest-9SF have clinical value since they can be discussed by the physician and the patient in choosing or adapting the care pathway.

The digital collection of Catquest-9SF data prior to consultations for patients with cataracts is implemented in routine practice in the two French ophthalmologic centres participating in this study (Institut Ophtalmologique Sourdille-Atlantique in Nantes and Chénieux Ophtalmologie in Limoges). There are many advantages to using notepads for data collection as these bypass the time-consuming step of transferring the data from paper to computer and makes it easy to improve the ergonomics. In both centres, patients completed the questionnaire in the waiting room using a notepad installed on a mobile table-top set which could be adjusted to the patient’s eye-level. Some elderly patients had found it difficult to click and move through the questions because they were using their nail on the touch screen, which caused them to click several times when entering an answer, causing mistakes and frustration. To improve the ergonomics, the table-top set was lowered, and the angle of the touch screen adjusted to facilitate clicking, which resulted in improved data quality and response rate. The electronic setup is also appropriate for use by clinicians as it presents each item’s raw score (not the Rasch analysed score) change from pre- to post-surgery using a spider chart. This simple dashboard with an ergonomic layout was found to be suitable for use in routine care.

Further work is needed to improve and adjust this layout and make it legible for patients. Another issue may be that being asked to complete the questionnaire before a decision is taken regarding surgery might tempt the patient to exaggerate their symptoms to get surgery. It is therefore important to ask for honest reporting if Catquest-9SF is to be used in establishing indications for surgery.

One weakness in the present study is the handling of postoperative questionnaires. The electronic notepads were situated at the participating clinics, indicating that the postoperative questionnaires could only be completed by those patients who made a postoperative clinic visit. This might have introduced a bias concerning follow-up data. Moreover, the answers given by patients completing the postoperative questionnaire at the clinic may have been influenced by a wish to please the surgeon.

This study has shown that Catquest-9SF is a suitable measure of subjective visual functioning in a French cataract population in France. The questionnaire was successfully translated into French and demonstrated robust psychometric properties. It is therefore suitable for use in French patients with cataract and following cataract surgery. Applicability in daily clinical practice may be improved without loss of accuracy by using a digital version of the questionnaire. This digital format alleviates the burden of data collection, improves data quality, and facilitates real-time discussions between practitioners and patients during consultation.

## Conclusions

A French version of the Catquest-9SF was presented and showed good psychometric properties. This version is suitable for use in French-speaking patients. The French version of Catquest-9SF was presented to patients through an electronic notepad. The electronic format of the Catquest-9SF was convenient for patients and showed good validity.

## Supplementary Information

**Additional file 1.** Patient workflow with the digital version of Catquest-9SF.

## Data Availability

The datasets generated and analysed during the current study are not publicly available due to integrity issues but are available from the corresponding author on reasonable request.
