# Supplementary material for: Validity of the French version of Catquest-9SF and use of an electronic notepad for entering patient-reported outcome measures
Source: Eye Vis (Lond). 2021 Apr 1;8:11. doi: 10.1186/s40662-021-00233-7 (PMC8015069; doi:10.1186/s40662-021-00233-7)
Supplement: Supplementary file 1 — Additional file 1. Patient workflow with the digital version of Catquest-9SF. [file 40662_2021_233_MOESM1_ESM.docx]

**Patient workflow with the digital version of Catquest-9SF**

The following workflow was used: (1) the patient was welcomed at the front desk of the clinic; (2) the patient was given a QR code; (3) in the waiting room, the patient was seated next to an electronic notepad with table-top support; (4) the patient flashed the QR code to access the questionnaire; (5) the patient received information and was invited to give consent; (6) the patient self-administered the nine questions (one question per screen); (7) the results were discussed during the consultation, and combined with clinical measures; (8) the surgeon analysed the pre and postoperative scores.
